# Switching from a high-fat cellulose diet to a high-fat pectin diet reverses certain obesity-related morbidities

**DOI:** 10.1186/s12986-018-0294-7

**Published:** 2018-08-06

**Authors:** Julie K. Bray, Gabriel S. Chiu, Leslie K. McNeil, Morgan L. Moon, Robyn Wall, Albert E. Towers, Gregory G. Freund

**Affiliations:** 1 Department of Pathology, Program in Integrative Immunology and Behavior, University of Illinois, Urbana, IL USA; 2 Division of Nutritional Sciences, University of Illinois, Urbana, IL USA; 3 Department of Animal Sciences, University of Illinois, Urbana, IL USA; 4 Department of Pathology, College of Medicine, University of Illinois at Urbana Champaign, 506 South Mathews Avenue, Urbana, IL 61801 USA

## Abstract

**Background:**

Reducing caloric intake is a proven intervention for mitigating and modulating morbidities associated with overnutrition. Caloric restriction is difficult to affect clinically, therefore, dietary interventions that ameliorate the adverse consequences of overnutrition in the presence of a high-calorie diet would be of value.

**Methods:**

Mice were fed an obesogenic diet containing 60% fat + 10% cellulose (HFC), or a control diet containing 10% fat + 10% cellulose (LFC) for 12 wks. Subgroups of mice were then switched from HFC to each of the following diets for an additional 5 wks: 1) 60% fat + 10% pectin (HFP), 2) LFC or 3) 10% fat + 10% pectin (LFP). To test for statistical differences, one-way or two-way ANOVAs were used with or without repeated measurements as needed.

**Results:**

In comparison to HFC, HFP prevented additional weight gain while LFC and LFP triggered weight loss of 22.2 and 25.4%, respectively. Mice continued on HFC experienced a weight increase of 26% during the same 5 wk. interval. After 12 wks, HFC decreased mouse locomotion by 18% when compared to control diet, but a diet switch to LFC or LFP restored mouse movement. Importantly, HFP, LFC, and LFP reduced fasting blood glucose when compared to HFC. Likewise, HFP, LFC and LFP improved glucose tolerance and decreased fatty liver by 37.9, 49.8, 53.6 and 20.2%, 37.2, 43.7%, respectively.

**Conclusions:**

Taken together, the results indicate that the dietary fiber pectin can mitigate some adverse consequences of overnutrition even in the presence of high-fat.

## Background

Obesity is a significant world-wide health problem that has more than doubled in incidence since 1980 [14, 25, 27]. According to the World Health Organization, obesity is projected to affect 1 in 5 people worldwide by 2025 [2]. Obesity is a critical risk factor in the development of type 2 diabetes [13, 24] and cancer [17, 71], likely tied to obesity-associated chronic inflammation, oxidative stress and hyperglycemia [16, 23, 28, 81]. Recently, obesity appears especially tied to non-alcoholic fatty liver disease (NAFLD) due, in a part, to high-fat diet-associated overnutrition [53, 85]. In clinical studies, the development of obesity is negatively associated with physical activity [62] and positively correlated with fat intake [32]. Therefore, interventions that mitigate the ability of the body to utilize dietary fat might forestall obesity.

Dietary fibers have been proposed as an intervention against obesity [68]. Rationale for their use includes a demonstrated ability in humans to reduce appetite, energy intake, and body weight [76, 80]. Dietary fibers (usually classified as soluble or insoluble) are generally carbohydrates from plants that resist digestion and absorption in the human small intestines [1]. Soluble fibers, such as gums, insulin-type fructans, and pectin, dissolve in water and are easily fermented by the microbiota of the large intestine [41]. Insoluble fibers, such as lignin and cellulose, do not dissolve in water and resist fermentation [41]. In terms of body weight regulation, both soluble and insoluble fibers can promote weight loss [33], but there is an inconsistent relationship between fiber solubility and its ability to reduce appetite and body weight ([20, 44], Isken 2010). While soluble fiber can promote weight loss [12], other factors such as coincident fat intake [57] and calories inherent to the short-chain fatty acid fermentation products [26, 40, 77] can negate this salutary effect.

The soluble dietary fiber pectin positively impacts blood glucose regulation [42, 65], sparking interest in its application to diabetes prevention/treatment [10, 37, 70]. Pectin is a polysaccharide that is primarily found in citrus peels and is often used as a gelling agent in the food industry [74]. Previously, we demonstrated that dietary pectin, when compared to the insoluble fiber cellulose, sped recovery from endotoxin in a mouse model of sepsis [64]. Since cellulose is the main structural component in plants [67], it is generally the most consumed of the dietary fibers [46, 50]. Pectin, in contrast, is consumed on a limited basis [46, 50] since it is a minority component of the edible parts of plants [63]. Previous studies have shown that pectin in conjunction with a HFD can reduce inflammation, cholesterol and liver fat [36, 78]. Therefore, we compared the effectiveness of high and low fat diets in conjunction with cellulose and pectin with regard to modifying the impact of HFD-induced obesity in mice.

## Methods

### Animals

Animal use was conducted in accordance with Institutional Animal Care and Use Committee approved protocols at the University of Illinois. C57BL/6 J male animals were purchased from Jackson Laboratory (Bar Harbor, ME) at 3 weeks of age. Mice were group housed (8 mice/cage) in standard shoebox cages (length 46.9 cm; width 25.4 cm; height 12.5 cm) and allowed water and food ad libitum. After 11 weeks on diet, mice were individually housed in small shoebox cages (length 28.9 cm; width 17.9 cm; height 12.7 cm) and acclimated for a week before switching to the new diets. Housing temperature (72 °F) and humidity (45–55%) were controlled as was a 12/12 h reversed dark-light cycle (2200–1000 h). Video recording of animal behavior was performed under red light using a Night Shot capable video camera (Sony HDR-XR500V). Except for body weight, food intake and locomotor activity, all treatments at all time-points represent separate cohorts of mice. The total number of mice utilized was 80.

### Diets

All diets were purchased from Research Diets (New Brunswik, NJ). Mice were fed open source uniform-base diets containing either 10% calories from fat with 100 g of cellulose (LFC, D06082201) or 60% calories from fat with 100 g of cellulose (HFC, D07102501) starting at 4 weeks of age. LFC-fed mice were kept on the same diet to serve as control. After 12 weeks on diet, mice fed HFC were switched to either LFC, 10% calories from fat with 105 g of pectin (LFP, D06082202), 60% of calories from fat with 105 g of pectin (HFP, D08111803), or HFC (Table 1).

**Table 1:** Nutrient composition of LFC, LFP, HFC and HFP

	D06082201 (LFC)		D06082202 (LFP)		D07102501 (HFC)		D08111803 HFP	
	Weight (g)	Kcal (%)	Weight (g)	Kcal (%)	Weight (g)	Kcal (%)	Weight (g)	Kcal (%)
Protein	18.4	20	18.3	20	24.6	20	24.5	20
Carbs	64.3	70	64.0	70	24.7	20	24.6	20
Fat	4.1	10	4.1	10	32.8	60	32.6	60
Total	86.7	100	86.3	100	82.1	100	81.7	100
Kcal/g	3.67		3.65		4.92		4.89	
Pectin	0		105		0		105	
Cellulose	100		0		100		0	

### Body weight

Mice were weighed weekly using the Adventurer Pro digital scale (Ohaus, Parisippany, NJ) as a repeated measure over time.

### Food intake

Food intake was measured in individually-housed mice, as a repeated measure, as we have described [75]. In brief, food was moved from the overhead cage food hopper and placed in an 8 cm diameter × 5 cm stainless steel bowl in conjunction with replacing the bedding. After 24 h, food intake was considered to be the difference between the weight of the bowl plus food at the beginning of the 24 h period and the weight of the bowl plus food and food recovered from the bedding after the 24 h period.

### Locomotion

Spontaneous locomotor activity was measured, as a repeated measure, as we have described [11, 45, 87]. Mice were video recorded in their home cage for 5 min. Distance moved was quantified using EthoVision XT 7.

### Glucose testing

Blood glucose was measured as we have previously described [45] in mice that had fasted for 16 h. For glucose tolerance testing, mice were injected i.p. with a 20% glucose solution equaling 1% of total body weight. Mouse tail blood glucose was recorded using a FreeStyle Freedom blood glucose monitor (Abbott, Abbott Park, IL) after the tail was cleaned with 70% ethanol and lanced with a sterile 18-gauge hypodermic needle (Franklin Lakes, NJ). Glucose was measured every 15 min for 90 min then every 30 min until 180 min post-injection. Results represent the total area under the curve of blood glucose versus time as calculated using the trapezoidal rule [72].

### Histochemistry

As we have previously described [11, 38], mice were euthanized by CO_2_ asphyxiation. The mouse chest cavity was opened and the right atrium incised. Mice were perfused through the left ventricle with 30 mLs of ice cold PBS using a 18 gauge needle and 30 mL syringe. Mice were next perfused with 30 mLs of ice-cold 10% neutral buffered formalin. Harvested livers were additionally fixed in 10% neutral buffered formalin for 24 h. Livers were subsequently paraffin embedded and sectioned. Five micron section was stained with hematoxylin and eosin (H&E) using an automated stainer (Sakura). Stained slides were imaged at 40× with a NanoZoomer 2.0-HT (Hamamatsu, Bridgewater, NJ). Percent of liver fat was determined using ImageJ by methods provided previously described (NIH) [3].

### Statistics

All data are presented as mean ± SEM. Data was analyzed using Sigma Plot 11.2 (Systat Software, Chicago, IL). To test for statistical differences, a one-way or two-way ANOVA was used with or without repeated measurements where needed. Tukey’s test was used for post-hoc pair-wise multiple comparison procedures. Where indicated, raw data was transformed using a log10 transformation to attain equal variance. All statistical analysis included testing for time point x treatment interactions. Statistical significance was denoted at *p* < 0.05.

## Results

### Switching from HFC to HFP prevents weight gain, while switching to LFC or LFP causes weight loss

To determine the effects of fat and fiber intake on diet-induced obesity, mice were fed a HFC diet for 12 weeks (baseline) then either maintained on the HFC diet or switched to HFP, LFC or LFP. As a control, a concurrent group of mice were fed LFC. Mice fed HFC for 12 weeks weighed significantly more than mice fed LFC (Table 2, *p* < 0.001). After the diet switch, HFC mice gain weight (Table 2, time effect of HFC, *p* < 0.001) while mice switched to HFP did not gain weight (Table 2, time effect of HFP, *p* = 0.42). In contrast, mice switched to either LFC or LFP lost weight (Table 2, time effect of LFC, *p* < 0.001, time effect of LFP, *p* < 0.001). Interestingly, by 1 week post-diet switch, mice demonstrated weight loss (Tables 2, 1 wk. post-switch: main effect of fat, *p* < 0.001, fiber, *p* = 0.01 and interaction, *p* = 0.29). In addition, the weight modulating effects of pectin and low fat diet were maintained for 5 weeks after diet switching (Tables 2) 2 wk. post-switch: main effect of fat, *p* < 0.001, fiber, *p* = 0.002 and interaction, *p* = 0.13. 3 wk. post-switch: main effect of fat, *p* < 0.001, fiber, *p* < 0.001 and interaction, *p* = 0.01. 4 wk. post-switch: main effect of fat, *p* < 0.001, fiber, *p* < 0.001 and interaction, *p* = 0.003. 5 wk. post-switch: main effect of fat, *p* < 0.001, fiber, *p* < 0.001, and interaction, *p* = 0.007). Finally, LFC control was compared to all diets at 1–5 weeks after diet switching (Table 2). HFP and HFC diets were significantly different than LFC at all weeks measured, and LFC was significantly different only at 3 weeks post-switch. Table 3 demonstrates food intake.

**Table 2:** Body weight of mice switched from HFC to LFC, LFP, HFP and HFC

Starting Diet	Baseline Body Weight (g)	Switch Diet	Weeks Post-Switch Body Weight (g)				
			1	2	3	4	5
LFC	23.99 ± 0.30	–	26.54 ± 0.39	26.94 ± 0.40	26.98 ± 0.45	27.29 ± 0.53	27.02 ± 0.52
HFC	37.29 ± 0.62^#^	LFP	27.54 ± 0.89^c^	26.92 ± 0.59^c^	28.00 ± 0.31^c^	28.88 ± 0.44^c^	27.81 ± 0.42^c^
		LFC	29.53 ± 1.18^c^	28.78 ± 0.97^c^	29.11 ± 0.76^#,c^	29.48 ± 1.05^c^	29.00 ± 1.07^c^
		HFP	35.22 ± 1.34^#,a^	35.41 ± 1.17^#,a^	35.94 ± 1.16^#,a^	36.96 ± 1.37^#,a^	37.93 ± 1.48^#,a^
		HFC	39.89 ± 1.47^#,b^	42.20 ± 1.73^#,b^	44.35 ± 1.78^#,b^	46.28 ± 1.96^#,b^	46.98 ± 1.98^#,b^

Mice were fed HFC as the starting diet for 12 weeks then switched to LFP, LFC, HFP, or HFC. Mice fed LFC as the starting diet were maintained on LFC as control. Results are expressed as mean ± s.e.m.; *n* = 10–11. Points without a common superscript are different (*p* < 0.05). # indicates significance versus LFC control (*p* < 0.05)

**Table 3:** Food intake of mice switched from HFC to LFC, LFP, HFP and HFC

Starting Diet	Switch Diet	Weeks Post-Switch Food Intake (g)				
		Diet Switch	1	2	3	4
LFC Control	–	3.7 ± 0.1^a^	3.2 ± 0.2^a^	3.6 ± 0.2^a,c^	4.0 ± 0.2	3.4 ± 0.4
HFC	LFP	0.4 ± 0.1^b^	0.8 ± 0.2^b^	3.4 ± 0.1^b^	3.6 ± 0.3	3.0 ± 0.1
	LFC	0.6 ± 0.2^b^	1.5 ± 0.4^b^	4.1 ± 0.1^c^	3.7 ± 0.2	3.2 ± 0.2
	HFP	4.3 ± 0.4^c^	4.2 ± 0.6^a^	4.0 ± 0.2^a,c^	4.1 ± 0.3	4.3 ± 0.9
	HFC	3.7 ± 0.1^a,c^	3.2 ± 0.2^a^	3.6 ± 0.2^a,b^	4.0 ± 0.2	3.4 ± 0.4

Mice were fed a LFC or HFC diet for 12 weeks (baseline) then either maintained on the LFC (LFC control) or switched from HFC to LFP, LFC, HFP, or HFC diets. Weekly measurements of daily food intake were collected each week post-switch for 4 weeks. Results are expressed as means ± s.e.m.; *n* = 5–13. Points without a common superscript are different (*P* < 0.05)

### A HFC diet reduces locomotor activity which only a LFC or LFP diet corrects

To compare the impact of LFC and HFC diets on baseline voluntary physical activity, locomotion was examined after 12 weeks (Table 4). To see if a dietary switch could improve locomotor activity of mice fed -HFC for 12 weeks, mice were switched to HFP, LFC or LFP for 3 weeks (Table 4, main effects of fat, *p* < 0.001, fiber, *p* = 0.94 and interactions, *p* = 0.39). At 3 weeks post-switch, HFP and HFC diets had significantly reduced locomotor activity compared to all low-fat diets. Table 4 also shows that the salutary effect of a low-fat diet on locomotor activity was present at 5 weeks (main effects of fat, *p* < 0.001, fiber, *p* = 0.19 and interactions, *p* = 0.59).

**Table 4:** Impact on locomotion of switching from HFC to LFC, LFP, HFP and HFC

Starting Diet	Baseline	Switch Diet	Weeks Post-Switch Locomotor Activity (cm)	
			3	5
LFC	1502.66 ± 80.20^a^	–	1533.83 ± 111.39	1834.80 ± 133.71
HFC	1232.45 ± 56.27^b^	LFP	1470.25 ± 68.30^a^	1818.48 ± 103.93^a^
		LFC	1404.95 ± 90.70^a^	1904.84 ± 102.28^a^
		HFP	924.76 ± 86.23^b,#^	1136.88 ± 132.45^b,#^
		HFC	1001.79 ± 81.53^b,#^	1340.66 ± 79.74^b,#^

Mice were fed HFC as the starting diet for 12 weeks then switched to LFP, LFC, HFP, or HFC. Mice fed LFC as the starting diet were maintained on LFC as control. Results are expressed as mean ± s.e.m.; *n* = 10–11. Points without a common superscript are different within each week post-switch (*p* < 0.05). # indicates significance versus LFC control within each week post switch (*p* < 0.05)

### Switching from HFC to HFP, LFC, or LFP improves fasted blood glucose levels and blood glucose tolerance

**Fig. 1:**
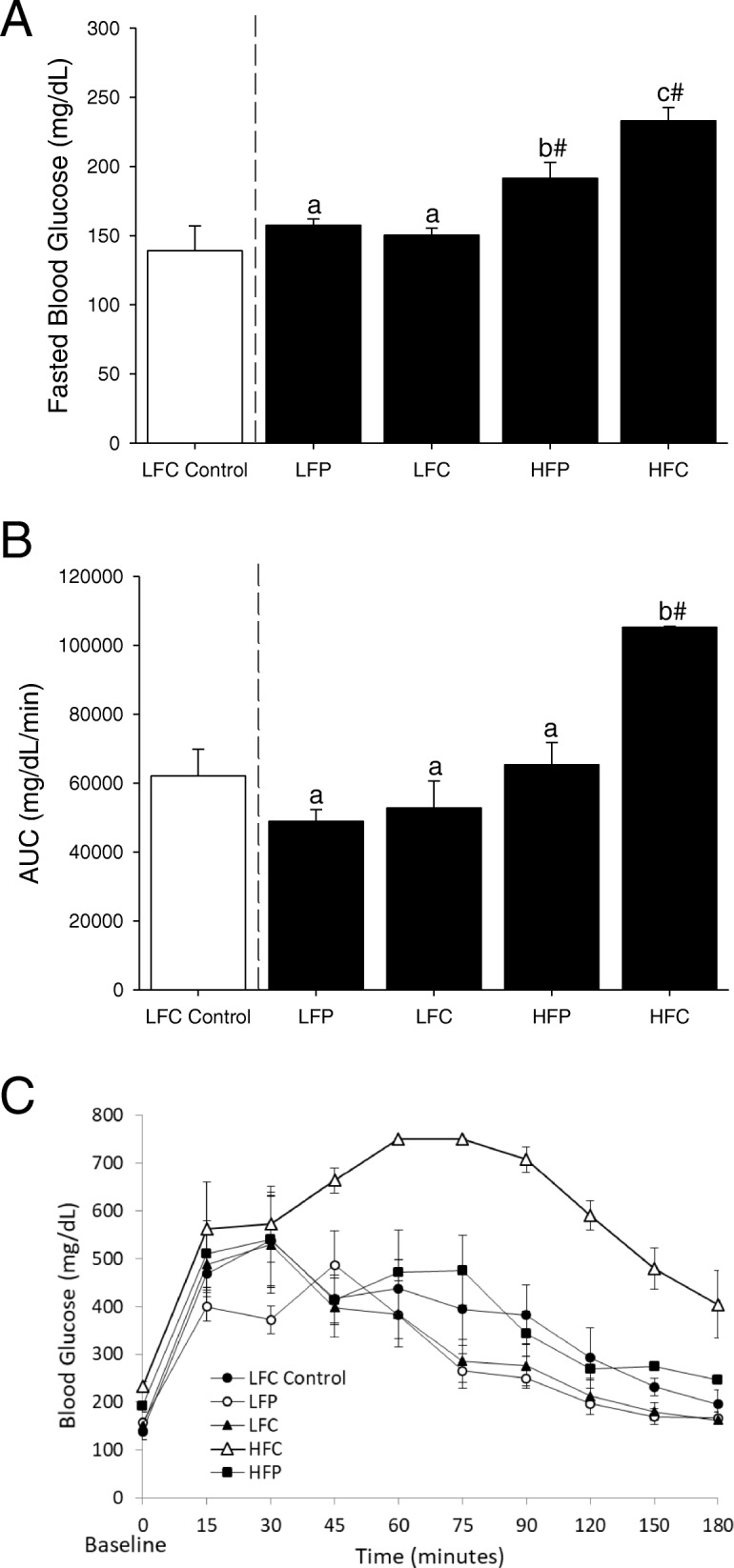
Switching from HFC to HFP, LFC, or LFP improves fasted blood glucose levels and blood glucose tolerance*.* (**a**) Mice were fed either LFC or HFC for 12 weeks and then HFC fed mice were switched to HFP, LFC or LFP for 4 weeks. Fasted (16 h) blood glucose was measured. Results are expressed as means ± s.e.m.; *n* = 3. Points without a common superscript are different (*p* < 0.05). # indicates significance versus LFC control (*p* < 0.05). (**b**) Mice were fed either LFC or HFC for 12 weeks and then HFC fed mice were switched to HFP, LFC or LFP for 4 weeks. Intraperitoneal glucose tolerance was measured over 180 min. Results are expressed as means ± s.e.m. of area under the curve (AUC) of blood glucose versus time; *n* = 3. (**c**) Blood glucose levels from mice treated in (**b**). Results are expressed as means ± s.e.m. of mg/dL of blood glucose; *n* = 3. Points without a common superscript are different (*p* < 0.05). # indicates significance versus LFC control (*p* < 0.05)

Fasting blood glucose was examined in mice that were fed a HFC diet for 12 weeks then switched to HFP, LFC, or LFP for 4 weeks. Mice switched to LFC or LFP had similar blood glucose levels compared to LFC control, while mice switched to HFP displayed lower blood glucose level than those fed the HFC diet (Fig. 1a, main effect of fat, *p* = < 0.001, fiber, *p* = 0.10 and interactions, *p* = 0.03; HFC v HFP, 233.00 ± 9.64 mg/dL v 191.33 ± 11.62 mg/dL, *p* = 0.01; HFC v LFC, 233.00 ± 9.64 mg/dL v 150.33 ± 5.04 mg/dL, *p* < 0.001; HFP v LFP, 191.33 ± 11.62 mg/dL v 157.50 ± 4.50 mg/dL, *p* = 0.04; LFC v LFP, 150.33 ± 5.04 mg/dL v 157.50 ± 4.50 mg/dL, *p* = 0.62; HFC v LFC control, 233.00 ± 9.64 mg/dL v 139.00 ± 18.01 mg/dL, p < 0.001; HFP v LFC control, 191.33 ± 11.62 mg/dL v 139.00 ± 18.01 mg/dL, p = 0.03; LFC v LFC control, 150.33 ± 5.04 mg/dL v 139.00 ± 18.01 mg/dL, *p* = 0.50; LFP v LFC control, 157.50 ± 4.50 mg/dL v 139.00 ± 18.01 mg/dL, *p* = 0.55). Fig 1b demonstrates the impact of diet switch on glucose tolerance (main effect of fat, *p* < 0.001, fiber, p = 0.01 and interactions, *p* = 0.02; HFC v HFP, 105290.00 ± 252.39 mg/dL/min v 65,360.00 ± 6386.17 mg/dL/min, *p* = 0.002; HFC v LFC, 105290.00 ± 252.39 mg/dL/min v 52,820.00 ± 7827.08 mg/dL/min, *p* < 0.001; HFP v LFP, 65360.00 ± 6386.17 mg/dL/min v 48,896.25 ± 3483.75 mg/dL/min, *p* = 0.10; LFC v LFP, 52820.00 ± 7827.09 mg/dL/min v 48,896.25 ± 3483.75 mg/dL/min, *p* = 0.67; HFC v LFC control, 105,290.00 ± 252.39 mg/dL/min v 62,157.50 ± 7695.9239 mg/dL/min, *p* = 0.003; HFP v LFC control 65,360.00 ± 6386.17 mg/dL/min v 62,157.50 ± 7695.92 mg/dL/min, *p* = 0.717; LFC v LFC control, 52,820.00 ± 7827.08 mg/dL/min v 62,157.50 ± 7695.92 mg/dL/min, *p* = 0.52; LFP v LFC control, 48,896.25 ± 3483.75 mg/dL/min v 62,157.50 ± 7695.92 mg/dL/min, *p* = 0.49). Fig. 1c shows the blood glucose levels during the 180 min glucose tolerance test.

### Switching from HFC to HFP, LFC, or LFP decreases liver adiposity

**Fig. 2:**
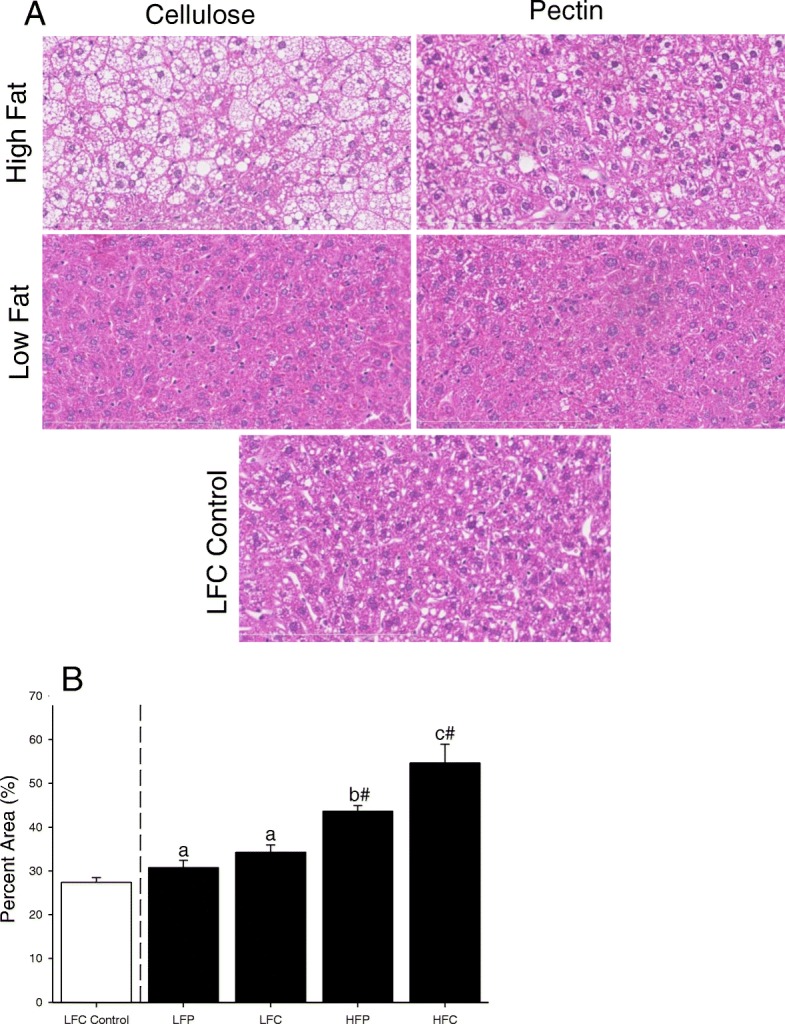
Switching from HFC to HFP, LFC, or LFP decreased liver adiposity*.* Mice were fed either LFC or HFC for 12 weeks and then HFC fed mice were switched to HFP, LFC or LFP for 5 weeks. (**a**) Liver histology H&E, representative, *n* = 3. (**b**) Liver adiposity quantified by image analysis. Results are expressed as means ± s.e.m.; n = 3. Points without a common superscript are different (*p* < 0.05). # indicates significance versus LFC control (*p* < 0.05)

Mice fed a HFC diet for 12 weeks were switched to HFP, LFC, or LFP for 5 weeks (Fig. 2a and b, main effects of fat, *p* < 0.001, fiber, *p* = 0.02 and interactions, *p* = 0.18; HFC v HFP, 54.65 ± 4.27% v 43.63 ± 1.32%, p = 0.02; HFC v LFC, 54.65 ± 4.27% v 34.30 ± 1.61%, *p* < 0.001; HFP v LFP, 43.63 ± 1.32% v 30.75 ± 1.65%, *p* = 0.01; LFC v LFP, 34.30 ± 1.61% v 1.32 v 30.75 ± 1.65%, *p* = 0.35; HFC v LFC control, 54.65 ± 4.27% v 27.37 ± 1.12%, *p* < 0.001; HFP v LFC control, 43.63 ± 1.32% v 27.37 ± 1.12%, *p* < 0.001; LFC v LFC control, 34.30 ± 1.61% v 27.37 ± 1.12%, *p* = 0.06; LFP v LFC control 30.75 ± 1.65% v 27.37 ± 1.12%, *p* = 0.32).

## Discussion

From 1850 to 1980, average life expectancy nearly doubled, increasing from about 39 years to about 75 years [60]. However, as the incidence of obesity accelerated in the 1970s, the rate of increased life expectancy slowed [6]. Some proposed that adverse health concerns associated with the obese state may be responsible for a proportion of decelerating average life expectancy [47, 56]. Clearly, obesity-linked co-morbidities like coronary heart disease, high blood pressure, stroke, type 2 diabetes, NAFLD and cancer support this contention [4]. To combat overnutrition-associated obesity, lifestyle changes such as calorie restriction and increased physical activity are advocated [52, 58], but these interventions are often of limited value outside the clinical setting [79]. For example, over 80% of people who intentionally lose 10 + % of body weight regain said weight within one year [82, 83]. Some have compared battling obesity with the treatment of chronic disease [51], where initial weight loss interventions require a commitment to maintenance therapies [22]. Therefore, mediations that reduce weight and are realizable long term have clinical significance.

Overnutrition can trigger fat cell proliferation and differentiation [43, 73]. Thus, in obesity, fat cells are not only larger but more numerous [7]. Interestingly, this change in fat cell physiology is associated with a decrease in the ability of mammals to lose weight [30, 49]. The high-fat diet (HFD) diet fed mouse used in this and many other studies [9, 61] is a well validated model of obesity and exemplifies many aspects of overnutrition-dependent human weight gain including the predisposition to NAFLD and glucose dyshomeostasis. Switching mice to a low-fat diet, regardless of fiber solubility, promoted over a 30% weight loss within one wk. Interestingly, this weight loss aligned mice previously fed a HFC for 12 wks with mice fed a LFC for 12 wks, indicating that these mice did not defend a HFD-induced weight gain as some animals and humans do [9]. These results also underscore the importance of decreased fat intake as an important way to reverse diet-induced obesity [39]. They also stress that dietary additives, like fiber, are no substitute for decreased caloric intake. Surprisingly, the HFP diet did promote weight loss and slow weight gain when compared to the HFC diet, suggesting that a diet high in soluble fiber may offset the weight-promoting effects of fat-based overnutrition.

Overall, obese mice demonstrate reduced spontaneous locomotion [8, 54]. Furthermore, some dietary fibers are associated with improved physical performance of certain motor tasks [84]. As expected, mice fed HFC were less mobile than mice fed LFC. Like with weight, HFD-fed mice given a low fat diet for 3 wks returned to “baseline” locomotion when compared to mice fed solely a low-fat diet for 15 wks. Unlike weight, HFP did not demonstrate a normalizing effect because these mice continued to match their HFC counterparts in showing reduced spontaneous locomotion. In addition, weight loss did not appear to correlate linearly with restored locomotion. These results suggest that the weight loss due to pectin intake is not a consequence of increased physical activity but likely due to a reduction in the bioavailability or utilization of energy. In general, the mechanisms by which dietary fiber can contribute to weight loss include: delaying gastric emptying, reducing glucose diffusion and preventing fat absorption [15, 18, 55]. Thus, increasing intake of dietary soluble fiber may be an effective means to reduce energy accessibility/metabolism either as a monotherapy or as an adjuvant intervention with calorie restriction and/or increased caloric expenditure [44, 68].

The relationship between dietary fiber and diabetes has been extensively studied [35, 86]. Many reports indicate that soluble fiber can significantly improve glycemic control for individuals with type 2 diabetes [66, 69]. To this point, Adam et al. demonstrated that soluble fiber in combination with a HFD can positively also impact lipidaemia and insulinaemia [5]. In general, the impact of fiber on blood sugar depends on type and dose. In mice, for instance, 10% psyllium and 10% sugar cane fiber decreased fasting blood glucose when added to an HFD for 12 weeks as did β-Glucan [41]. In humans, however, muffins high in β-glucan and resistant starch lowered blood glucose more effectively than muffins containing low or medium β-glucan/resistant starch [41]. Overall, dietary fiber is thought to reduce blood glucose through an increase in satiety via increased mastication, calorie displacement, and decreased absorption of macronutrients [41]. Viscous fibers like pectin form gels in the intestine which appear to limit the interaction between luminal contents and digestive enzymes while thickening the unstirred water layer decreasing the diffusion and uptake of glucose [41]. As shown here, pectin (in the presence of high-fat) can lower fasting blood glucose when compared to cellulose. Despite the above, how pectin improves glucose homeostasis is still not clear, but appears partially delinked from weight regulation. As the AUC data show, HFP completely normalized glucose clearance when compared to HFC.

When weight is evaluated, HFP mitigated fat-dependent gain by 50%. In general, dietary supplementation with fiber produces inconsistent results when weight loss is examined [20, 34, 41]. Again, gel formation in the intestine may be key to pectin-mediated reduced weight gain. The ability of gel-forming fibers, like pectin, to bind bile acids and micelle components, such as monoglycerides, free fatty acids and cholesterol decrease fat absorption in the gut [41]. Interestingly, NAFLD induced by a high-fat diet was also reduced when pectin replaced cellulose. Like weight, pectin appeared to mitigate fatty liver by about 50%. Fermentable fibers like pectin can impact the expression of acetyl-CoA carboxylase which is the rate-limiting enzyme in lipogenesis [41]. As example, a 10 wk. course of *Plantago ovata* increased AMPK phosphorylation reducing activity of acetyl-CoA carboxylase in obese rats to that in lean rats [29]. Additionally, short chain fatty acids due to gut fermentation of fermentable fibers appear to activate hepatic AMPK [41]. Such findings support the important role of soluble when compared to insoluble fiber when attempting to use diet as a first line treatment for pre-diabetes [19, 48], but underscores its limitation as a weight loss therapy.

## Conclusion

In humans, weight loss is difficult to realize and maintain [21]. Even effective procedural interventions like bariatric surgery often lose efficiency long-term [31, 59]. As shown here, palatable dietary interventions that can slow or prevent overnutrition-dependent weight gain are achievable in mice. Whether such methods can be translated to humans remains to be determined, since fermentable and gel-forming fibers may generate abdominal discomfort and oily/watery stools.

## Abbreviations

H&E: hematoxylin and eosin
HFC: 60% fat + 10% cellulose diet
HFP: 60% fat + 10% pectin diet
LFC: 10% fat + 10% cellulose diet
LFP: 10% fat + 10% pectin diet
